# Assessment of anthropogenic activities impact based on metals in soil and tree leaves along roadside in Bangladesh

**DOI:** 10.1038/s41598-025-91683-9

**Published:** 2025-02-26

**Authors:** Armin Anwar, A. H.M. Mezbah Uddin, Md. Siddiq Hasan, Md. Sohel Parvez, Bianka Sipos, Dina Bibi, Zsófi Sajtos, Béla Tótmérész, Tibor Magura, Edina Simon

**Affiliations:** 1 Department of Environmental Science and Management, North South University, Dhaka, Bangladesh; 2 Department of Botany, University of Dhaka, 1000 Dhaka, Bangladesh; 3 Department of Hydrobiology, Faculty of Science and Technology, University of Debrecen, Debrecen, 4032 Hungary; 4 Department of Oceanography, Noakhali Science and Technology University, Noakhali, 3814 Bangladesh; 5 Department of Ecology, Faculty of Science and Technology, University of Debrecen, Debrecen, 4032 Hungary; 6 HUN-REN–UD Anthropocene Ecology Research Group, Egyetem square 1, Debrecen, H-4032 Hungary; 7 Environmental Analytical Research Group, Department of Inorganic and Analytical Chemistry, Faculty of Science and Technology, University of Debrecen, Egyetem Square 1, Debrecen, H-4032 Hungary; 8 HUN-REN-UD Functional and Restoration Ecology Research Research Group, Egyetem square 1, Debrecen, H-4032 Hungary

**Keywords:** Bioindicators, *Ficus benghalensis*, Environmental health, Industrial pollution, *Polyalthia longifolia*, Road traffic pollution, *Swietenia macrophylla*, Plant sciences, Environmental sciences

## Abstract

The assessment of heavy metal pollution in leaves and soils is essential in environmental health monitoring. We assessed the effect of anthropogenic activities along an industrial–urban–rural gradient in the roadside soil and tree leaves of Bangladesh based on their metal concentration. The concentrations of Pb, Cr, Ni, and Cd were analysed in leaves of *Ficus benghalensis*, *Swietenia macrophylla*, and *Polyalthia longifolia* and topsoil samples using ICP-OES. We used pollution indices to quantify the impact of contaminants on ecosystems. Our findings indicated that there were higher metal concentrations, especially Cd and Pb, in soil and leaves in close proximity to locations with substantial traffic and industrial activities. Igeo index indicated moderately to strongly polluted areas by Cd. The contamination factor (CF) for Cd indicated a severe contamination, while moderately contamination show by Cr, Pb, and Ni. The value of the ecological risk factor was below 40 for Cr, Ni, and Pb; thus, negligible ecological risks were considered for these elements. But, the ecological risk assessment indices showed that Cd poses high ecological risks in soil and leaves across all study areas. Bioaccumulation analysis indicated substantial heavy metal absorption in tree foliage, with *F. benghalensis* and *P. longifolia* exhibiting elevated Pb accumulation in urban environments. The PCA indicated that Cd, Cr, Ni, and Pb mostly derived from anthropogenic sources in leaves and soil samples, too. Our results demonstrated that *F. benghalensis*, *S. macrophylla*, and *P. longifolia* are good indicator to assess the effect of anthropogenic activities on the terrestrial ecosystem. Our results also indicated the critical need for environmental monitoring and mitigation techniques in urban and industrial zones to reduce heavy metal pollution in roadside ecosystems.

**Supplementary Information:**

The online version contains supplementary material available at 10.1038/s41598-025-91683-9.

## Introduction

The rapid increase in industrialisation, unplanned urbanisation, heavy traffic, and extensive human activities leading to fast economic expansion has contributed to pollution in cities, particularly roadside areas, causing serious threats to the environment^1–3^. Consequently, various possibly harmful elements, particularly heavy metals, can accumulate in urban environments^1,2^. Heavy metals can rapidly accumulate in several environmental compartments, such as soil, air, and water, due to the growth of the human population. Roadside soils and dust serve as both sources and sinks for elemental contaminants from human activities in urban settings^3^. Roadside soil and dust deposited on surrounding tree leaves can be indicators of ambient air quality since they absorb diverse pollutants, such as heavy metals, from multiple sources^1^. Heavy metal concentrations in urban roadside environments threaten public health through ingestion, inhalation, and skin absorption^5,6^. Urban people, particularly children, are at serious risk for health problems because of the presence of Cr and Cd in street dust^4^. The carcinogenic hazards of Cr and As from ingesting and coming into touch with road dust could exceed the recommended thresholds^3^. Thus, studying the harmful elements in urban roadside environments and the health concerns they pose is crucial for maintaining the ecosystem, as well as the overall safety of city dwellers^7^.

The increasing use of fossil fuels in recent decades due to the emission of hazardous materials can cause acute and chronic adverse health effects on humans and animals. According to the International Agency for Research on Cancer (IARC), toxic heavy metals such as Cd, Pb, Cr and Ni are group one carcinogens. Exposure to these metals may lead to a decline in the mental, cognitive, and physical health^8^. Metals that are found in soil are a source of environmental concern because they remain in the soil for an extended period of time and have the potential to be exposed to humans, which in turn poses a threat to their health^9^. Soils are susceptible to environmental changes; therefore, the accumulation of potentially toxic elements in soils could be incorporated into the soil biogeochemical cycling and can be absorbed in plants by biological processes^10^. Furthermore, these substances induce a disturbance in the natural equilibrium of ecosystems due to their prolonged presence in soil and their significant accumulation in tree leaves^10^. In Bangladesh, urbanisation accompanied by a rise in vehicular emissions, large-scale infrastructure projects, and the expansion of various industries (such as brick kilns, alloy and steel manufacturing, and textile production), agrochemicals, and chemical fertilisers have contributed to environmental pollution. These activities contribute to the pollution of the atmosphere, which can be transported over considerable distances^11,12^. The potentially hazardous substances emitted from such intense human activities carried and spread over by atmospheric transportation can directly accumulate in roadside dust, soils, and tree canopies through deposition^2,13^. It is crucial to evaluate the harmful elements deposited in roadside soil and tree leaves in metropolitan areas and the associated health hazards to preserve the environment, ecology, and public health.

A lot of studies demonstrated that plants which were grown near an industrial area can accumulate metals in higher concentration than those from rural or other types of areas. Ahmad et al. (2010) studied the metal concentration in tomato, chili and cabbage, while Mottalib et al. (2016) analysed the metal concentration in spinach samples near Dhaka city^14,15^. Shaheen et al. (2016) demonstrated high metal concentration in different fruits and vegetables, such as banana, carrot, bean, tomato, green chili and potato^16^. Ahmed et al. (2016) demonstrated the metal accumulation in different fish species, as *Puntius ticto*,* Puntius sophore* and *Puntius chola* in Kamrangir Char and Amin Bazar in Dhaka^17^. Near Chittagong metal concentration of Poua, Chring, Rita and Chapila samples were analysed by Islam et al. (2013)^18^. Thus, earlier studies focused on the metal concentration analysis in fruits, vegetables and fish samples in the aspect of human health risk.

Tree leaves can also be used in biological monitoring to assess pollution because soil and vegetation are the sinks for metal deposition^19,20^. Plants can accumulate heavy metals in high concentrations, especially in the urban environment^19,20^. Nowadays, plant-based biomonitoring of environmental state has gained popularity, especially due to its cost-effectiveness and environmental friendliness, which are in contrast to traditional chemical and physical approaches. Based on their availability and affordability, urban plants are widely used for indirect monitoring of heavy metals in urban environments^15,16,20^. Many researchers all around the world study *Ficus benghalensis*^6,21,22^, *Swietenia macrophylla*^23,24^, and *Polyalthia longifolia*^6,21^. When combined, these approaches can be used to determine which areas are the most contaminated, allowing for the development of an effective environmental management plan.

Sultan et al. (2022) studied soil, dust, and leaf-based urban pollution from heavy metals and associated health threats^6^. Shahrukh et al. (2023) studied leaf-based indicators of metal buildup in Dhaka^21^. In both works, the selected study area and sampling were in the Dhaka city area. In the current study, we sampled roadside soils and tree leaves from different areas of Dhaka city and also from a rural village far from the city, representing undisturbed and pristine conditions for comparing city and rural scenarios. The ground water arsenic contamination is one of the most important public health problems in Bangladesh. But this arsenic pollution was associated with high geological background levels in Bangladesh^12^. Thus, we focused the analysis of metals in soil and tree leaves samples. The Cd, Cr, Ni and Pb analysis was chosen in our study because various anthropological activities (mining, agriculture, industry, traffic) can cause the emission these metals in high concentration in the environment. The objective of this study was to study the effect of anthropogenic activities based on metal concentrations in roadside soils and in tree leaves along urban, suburban, and rural gradients. To study the level of accumulation and the interaction between soil and plant leaves metal concentration geo-accumulation (Igeo) index, contamination factor (CF) and ecological risk factor (ERF) was calculated based on the metal concentration of soil and tree leaves. Our hypotheses are as follows: (i) there are significant differences in pollutants of soils and tree leaves areas along the urbanisation gradient, (ii) the highest metal concentrations in soil and leaves are in the urban and industrial environments, and (iii) high level of accumulation is found in the industrial areas for Cd, Cr, NI and Pb.

## Results and discussion

### Metal concentrations of roadside soil and leaves

The average metal concentrations of roadside soil for the studied areas are shown in Table 1. We found that the metallic concentrations of roadside soils exceeded the baseline levels established by the US EPA EcoSSL (Cd, 1 mg kg^− 1^; Cr, 7.5 mg kg^− 1^; Ni, 6.4 mg kg^− 1^; and Pb, 11.3 mg kg^− 1^; Table S1)^29,30^. The metal concentrations in soil along roadsides were higher than the metallic content in tree leaves along the roadsides (Table S1), which is consistent with previous studies in various areas of Dhaka city^6,25,26^.

**Table 1:** Concentrations of heavy metals (mean ± SD, mg kg^− 1^) in roadside soil samples in the study areas.

Studied sites	Pb	Cr	Ni	Cd
commercial	28.0 ± 11.8	21.6 ± 10.6	15.5 ± 5.3	6.3 ± 0.4
residential	29.1 ± 8.6	22.1 ± 5.3	17.3 ± 7.4	6.4 ± 0.3
industrial	31.4 ± 19.7	14.8 ± 8.9	11.3 ± 7.2	5.8 ± 3.0
rural	11.1 ± 3.1	17.1 ± 3	17.3 ± 3	6.1 ± 0.2

The average elemental concentrations found in the deposited dust on leaves are shown in Table 2. The average metallic concentrations of Cd and Pb in the roadside perennial tree leaves of all areas exceeded their permissible values^27^. The permissible values are as follows: Cd, 0.02 mg kg^− 1^; Cr, 1.3 mg kg^− 1^; Pb, 2 mg kg^− 1^; and Ni, 10 mg kg^− 1^. The average concentration of Pb in *F. benghalensis* leaves was higher than the concentrations in the leaves of *Ficus religiosa* of the urban, industrial, and rural areas of the Ayutthaya Province, Thailand^16^. The concentration of Pb found in the leaves of *P. longifolia* was lower than that from Lahore, Pakistan^28^. The average concentrations of Pb, Cr, Ni, and Cd found in the *Ficus* sp. and in *P. longifolia* were lower than the average elemental concentrations investigated related to other studies in Dhaka^6^.

**Table 2:** Concentration heavy metals (mean ± SD, mg kg^− 1^) in roadside tree leaves in the study sites.

Species	Cd	Cr	Ni	Pb
	commercial			
*F. benghalensis*	0.43 ± 0.1	1.64 ± 0.79	3.54 ± 3.23	11.38 ± 3.82
*P. longifolia*	0.33 ± 0.21	1.51 ± 0.87	2.71 ± 1.93	10.35 ± 8.63
*S. macrophylla*	0.38 ± 0.13	2.26 ± 1.09	4.41 ± 3.23	7 ± 2.98
	residential			
*P. longifolia*	0.3 ± 0.3	1.5 ± 0.4	1.65 ± 0.73	4.62 ± 5.8
*S. macrophylla*	0.3 ± 0	1 ± 0.1	1.58 ± 0.08	4.34 ± 1.07
	industrial			
*F. benghalensis*	0.3 ± 0.1	1.7 ± 1.1	1.55 ± 0.46	10.3 ± 6.72
*S. macrophylla*	0.4 ± 0.1	1.4 ± 0.1	5.26 ± 5.11	9.52 ± 0.81
	rural			
*F. benghalensis*	0.3 ± 0.1	1.5 ± 0.4	2.24 ± 1.02	8.83 ± 7.29
*P. longifolia*	0.3 ± 0.1	0.9 ± 0.4	2.84 ± 2.08	4.48 ± 3.86
*S. macrophylla*	0.2 ± 0.1	1.1 ± 0.4	1.17 ± 0.53	2.49 ± 0.5

We used a two-way analysis of variance (ANOVA) to explore the influence of geographical areas and tree species on the bioaccumulation capacity of heavy metals in the leaves of roadside trees (Table S2). We did not find statistically significant effects of area types on the concentration of Cd, Cr, and Ni in tree leaves. There were no statistically significant differences in metal bioaccumulation among the various tree species. However, the combined interaction between areas and tree species yielded statistically significant differences in Cd and Ni accumulation in roadside tree leaves. These findings emphasise the interplay between geographical areas and tree species in influencing heavy metal bioaccumulation patterns, particularly for Cd and Ni. This study contributes valuable insights into the complex dynamics of environmental factors impacting heavy metal distribution in urban tree ecosystems, i.e., the bioaccumulation of heavy metals in vegetation is accelerated by urbanisation and uncontrolled anthropogenic effects.

## Results of main component analysis of roadside soil and leaves

**Fig. 1:**
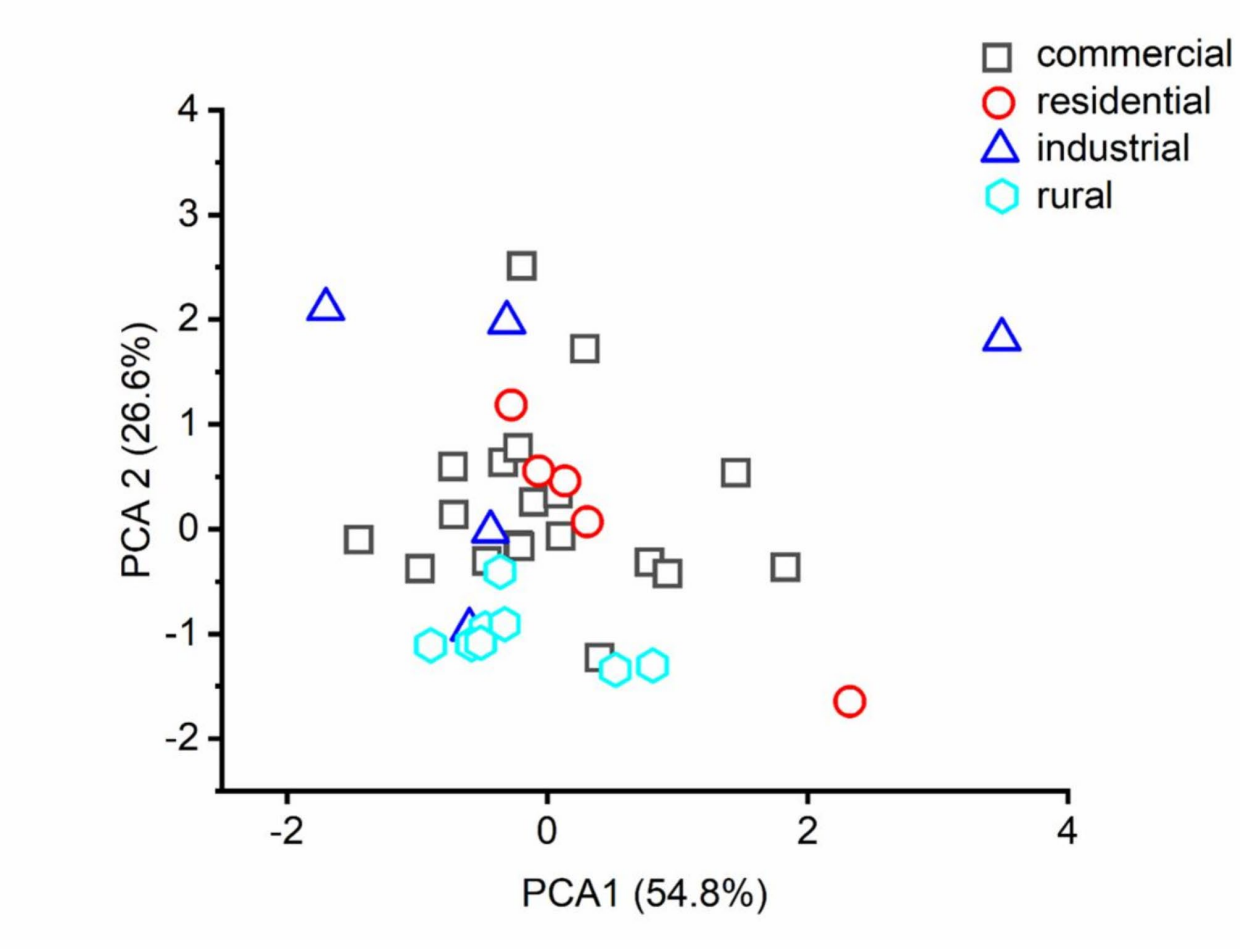
Principal component analysis (PCA) score plot based on metal concentrations in soil samples.

The first two principal components of the roadside topsoil data set explained 94.18% of the total variance. The first component (PC1) explained 63.16% of the total variance, and the loadings were dominated by Cd (0.031), Cr (0.12), Ni (0.009) and Pb (0.99). The second principal component (PC2) explained 31.018% of the variance and was dominated by Cd (0.067), Cr (0.88), Ni (0.45) and Pb (–0.11), respectively. In the principal component scatterplot, all the areas were overlapped with each other, suggesting that the potential sources of these heavy metals in soil might be the same, the high anthropogenic activities (Fig. 1).

**Fig. 2:**
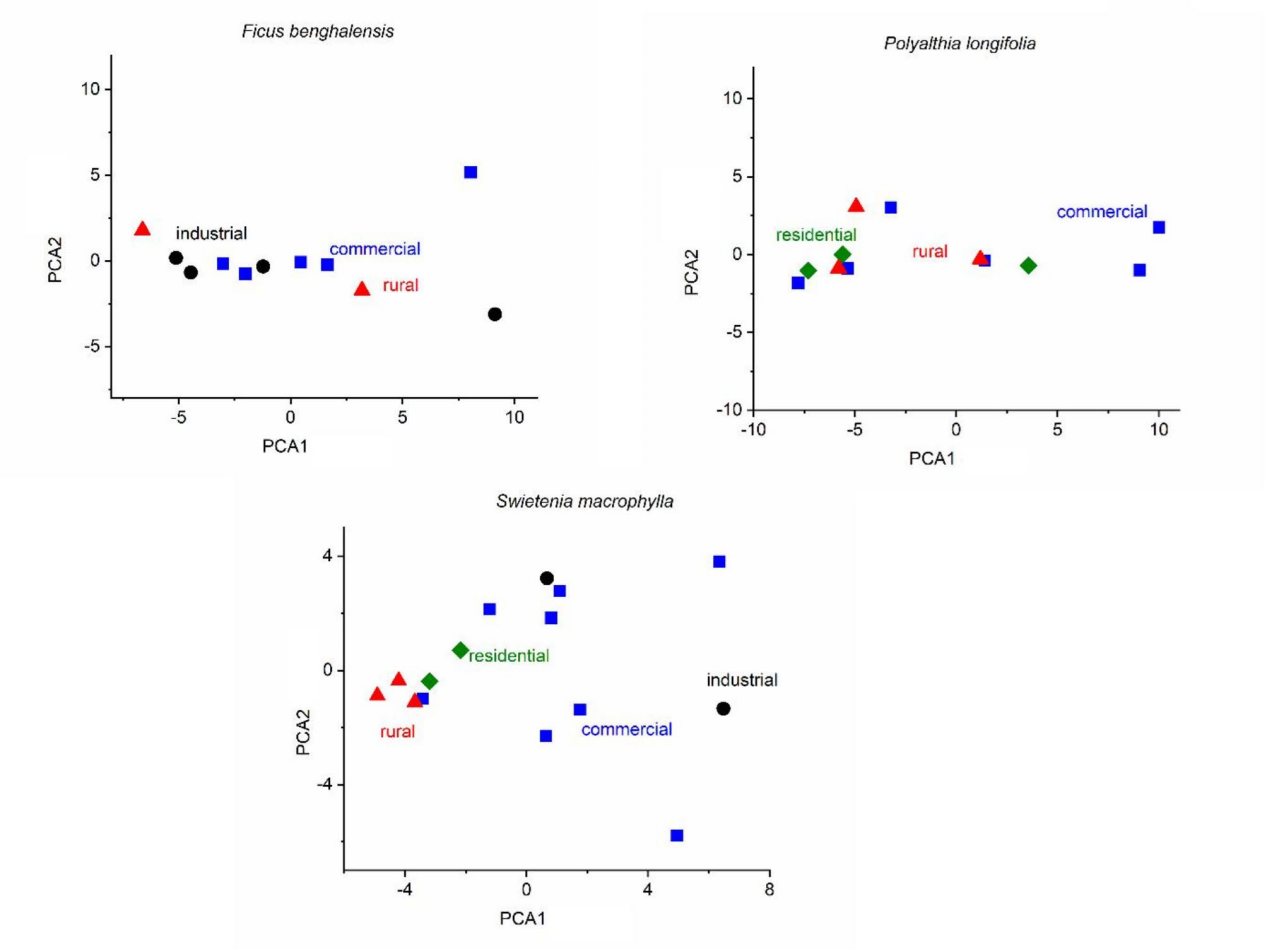
Principal component analysis (PCA) score plots based on metal concentrations in leaves of the studied species.

The first two components of the principal component analysis (PCA) of the elemental concentrations in *F. benghalensis* leaves explained 97.99% of the total variance (Fig. 2). PC1 was responsible for 84.106% of the variance and positively correlated with Pb (0.996), Cd (0.78), and Ni (0.44) and negatively correlated with Cr (–0.073). The PC2 explained 13.891% of the variance and positively correlated with Ni (0.9), Cd (0.35), and negatively correlated with Cr (–0.1) and Pb (–0.08). In *P. longifolia*, the two principal components (PC1 and PC2) explained 99.183% of the total variance.

PC1 was positively loaded with Pb (0.99), Cd (0.8), Ni (0.38), and Cr (0.22) and showed a total variance of 94.903%. PC2 was positively loaded with Ni (0.92), Cd (0.06), and Cr (0.03) and negatively loaded with Pb (–0.01) and showed a total variance of 4.28%. In *Swietenia macrophylla*, the rural area was well separated from the urban areas based on the elemental concentration of Cd, Cr, Ni, and Pb in leaves. A total variance of 97.62% occupied by the first two principal components greater than 1. The first principal component was responsible for 67.64% of the variance and was positively loaded by Pb (0.7), Ni (0.6), Cr (0.1) and Cd (0.02). The second principal component was responsible for 29.98% of the variance and was positively loaded by Pb (0.66), Cr (0.13) and negatively loaded by Ni (-0.7) and Cd (0.003). Overall, the sources of the studied four heavy metals, Cd, Cr, Pb, and Ni, in the tree leaves of *F. benghalensis*,* Polyalthia longifolia* and *Swietenia macrophylla* denoted the same possible contaminated sources, especially in the urban areas (Fig. 2).

## Results of indices

**Fig. 3:**
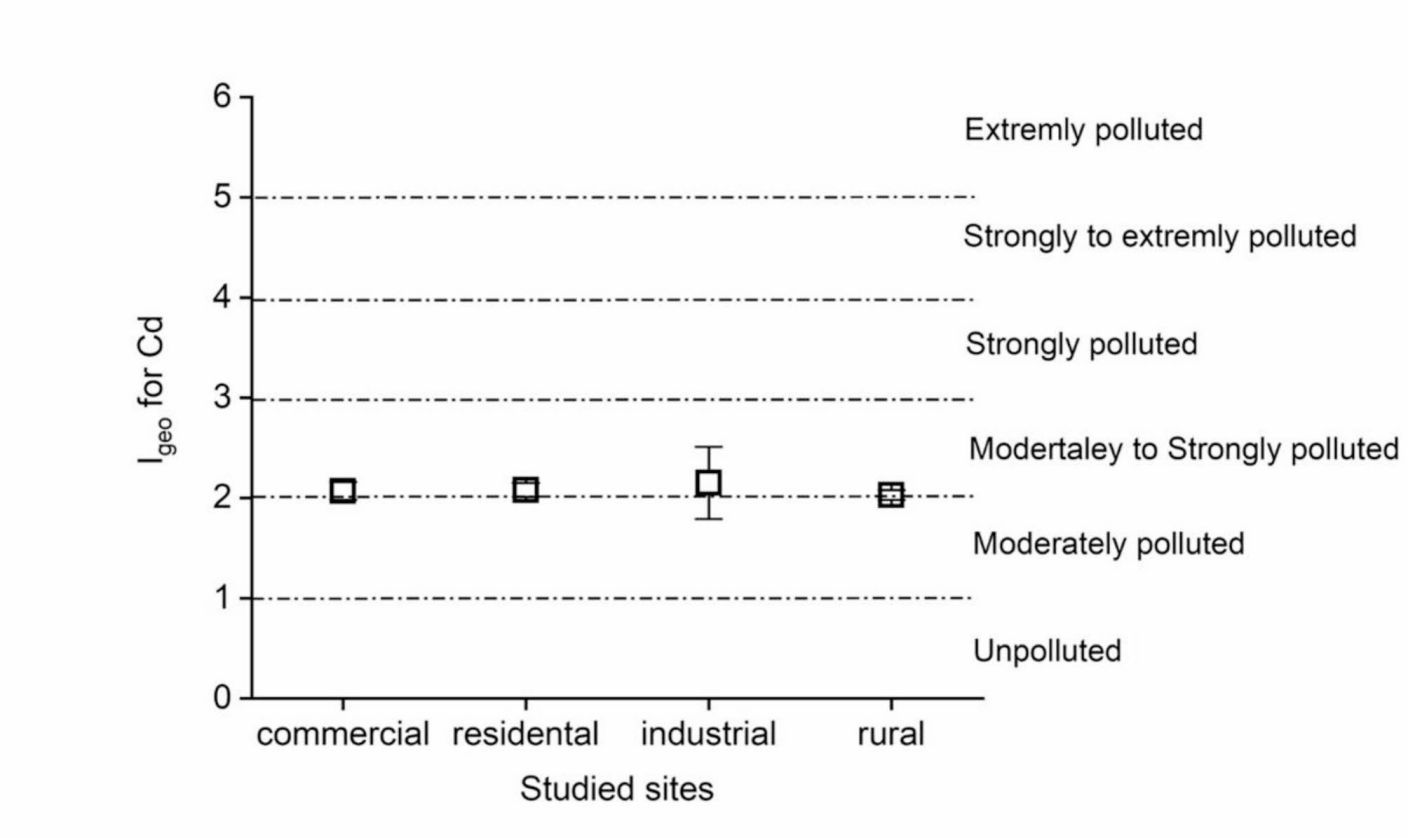
Igeo accumulation index for Cd in the different sites (mean ± SD).

Our findings suggest that based on the mean values of I_geo_ index, all the studied areas (i.e., urban commercial, urban residential, urban industrial and rural areas) were moderately to strongly polluted by Cd (Fig. 3, Table S3).

**Fig. 4:**
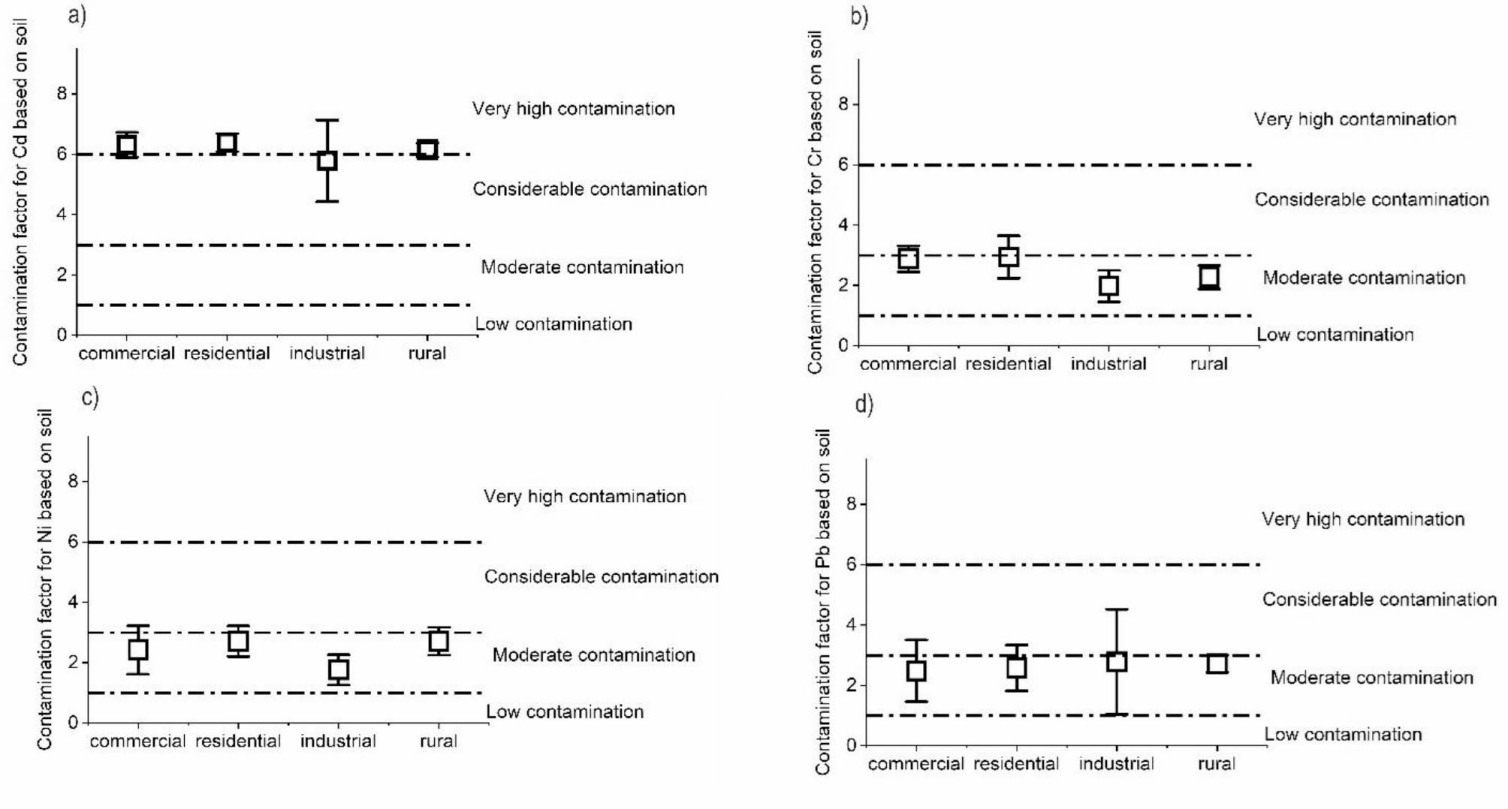
Contamination factors for Cd (a), Cr (b), Ni (c), and Pb (d) based on soil samples from the different sites (mean ± SD).

We found that the mean values of the contamination factor (CF) for Cd in the roadside soil were above 6, indicating severe contamination. The roadside soil was moderately contaminated by the three other heavy metals, Cr, Pb, and Ni (Fig. 4; Table S4**).**

**Fig. 5:**
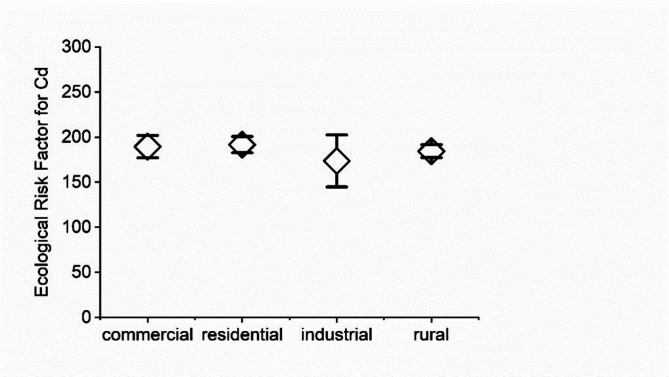
Ecological risk factor for Cd (mean ± SD).

The mean value of the ecological risk factor for the Cd was above 150 for all the studied locations (Table S5). The value of the ecological risk factor was below 40 for Cr, Ni, and Pb; thus, negligible ecological risks were considered for these elements. Cd was the predominant source of ecological risk, and all the sampling locations had extremely high ecological risk (ER > 160); more than 80% of the ecological risks originated from Cd (Fig. 5).

**Fig. 6:**
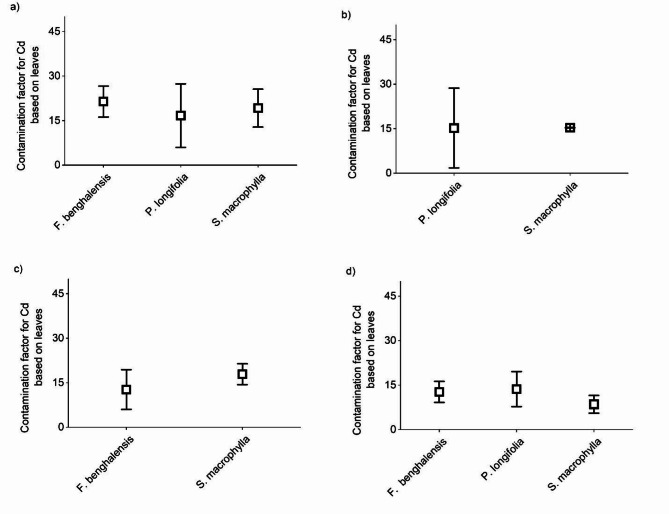
Contamination factor of metals based on leaves (mean ± SD) in commercial (a), residential (b), industrial (c), and rural sites (d).

We compared the CF of the elemental concentration of the roadside tree leaves to the WHO values^16^. We found that leaves of all the studied areas were severely contaminated by Cd (CF > 10) and considerably contaminated with Pb (3 ≤ CF < 6) (Fig. 6).

## Spatial distribution of trace elements

**Fig. 7:**
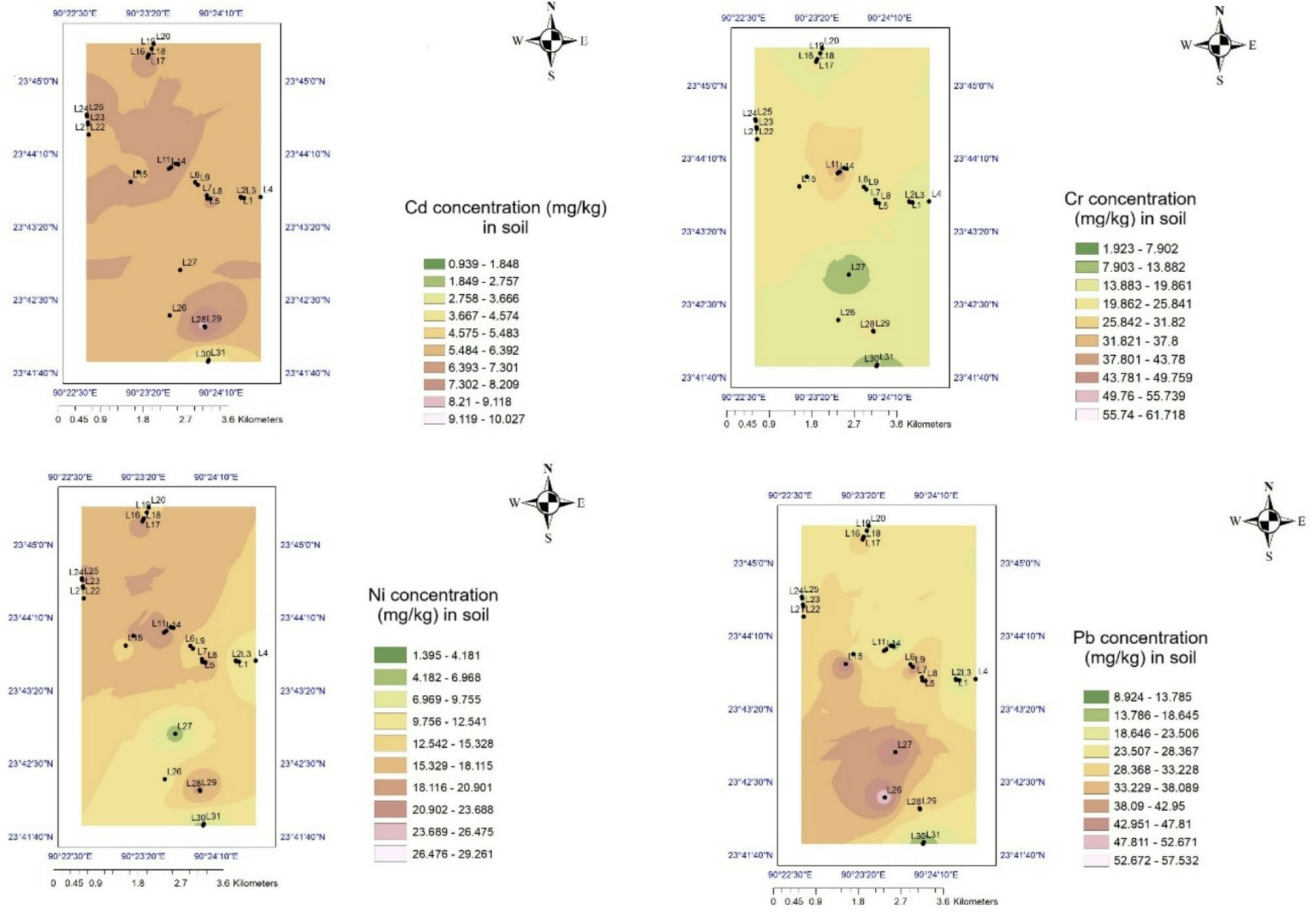
Spatial distribution pattern of heavy metals (Cd, Cr, Pb, and Ni) in roadside soil of Dhaka city (method: IDW interpolation, ArcGIS 10.8).

**Fig. 8:**
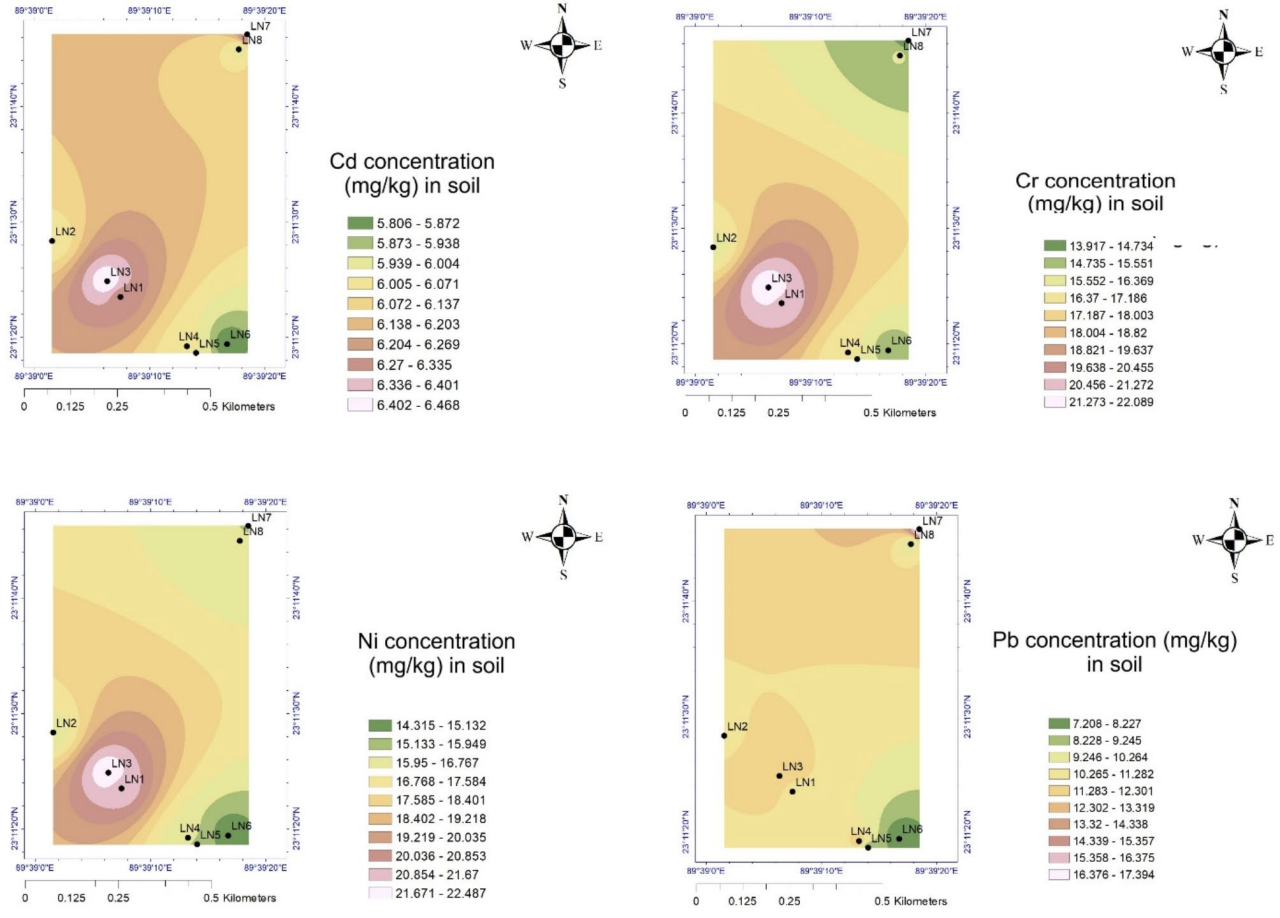
Spatial distribution pattern of heavy metals (Cd, Cr, Pb, and Ni) in roadside soil of rural area (method: IDW interpolation, ArcGIS 10.8).

The spatial distribution maps show the distribution patterns and concentration range of the heavy metals across the studied locations. To reveal the overall environmental pollution, it is crucial to understand the spatial distribution of heavy metals^32^. The spatial distribution of metal content in tree leaves is not uniform^6^. In the spatial distribution map, the red areas indicated the hotspots for the highest heavy metal concentrations in Dhaka city^4^. The Cd content was higher in all areas of Dhaka city (i.e., urban commercial, urban residential and urban industrial). The Pb distribution pattern was almost uniform in the rural areas, but a higher concentration of Ni and Pb was found in the northwest direction (i.e., in the urban commercial region) and in the industrial area (Figs. 7 and 8). Furthermore, anthropogenic sources of Cd include mining, smelting, wastewater irrigation, industrial emissions, manufacturing, motorised vehicles, electroplating, the use of Cd batteries, agrochemicals, fungicides etc. Every year, a variety of industries are established in the city of Dhaka, releasing metals into the atmosphere^8^. The main sources of Cd, Ni, Zn, Pb, Cu, Mn, Cr, etc., from these locations are traffic density, machinery, electroplating industries, chemicals from textile industries, alloys, and plastic manufacturing industries. Although the metallic concentration in the rural zone was found to be lower than the urban zone, maximum concentrations for Cd, Co, Cu, Cr, Mn, Ni, and Zn were found in the Poddar Para, Lohagara which is a mainly high-density residential and commercial area. Hence, the anthropogenic activities are higher, and the lowest concentrations were observed in the Sarkar Para and Kumar Kanda, which are calm residential areas bestowed with greenery and the least anthropogenic activities.

Cd is released from many sources, both natural and anthropogenic, and eventually accumulates in the soil and then bio-accumulates in vegetation. One of the main natural sources of Cd in soil is geological weathering. Furthermore, every year, a variety of industries are established in the city of Dhaka, releasing metals into the atmosphere^26^. The main sources of Cd, Ni, Pb and Cr from these locations are traffic density, machinery, electroplating industries, chemicals from textile industries, alloys, plastic manufacturing industries, Cd–Ni batteries, automobile tyres, brake abrasion, lubricants, solid wastes, uprising ceramics industries, agrochemicals, fungicides etc^25,33,34^. Cd may easily be released into the environment as a result of friction between the road and an automobile tyre. Cd can also be found in roadside soils due to diesel fuel and lubricating oil leaks^35^. Untreated industrial effluents and contaminated wastewater from the road’s surface, which has partly been deposited in the soil next to the road, also affect the soil beside the road^12,36,37^. Cd may easily be released into the environment as a result of friction between the road and an automobile tyre. Cd can also be found in roadside soils due to diesel fuel and lubricating oil leaks^38^. Contaminated wastewater from the road’s surface in Dhaka city, which has partly deposited in the soil next to the road, also affects the soil beside the road^36,37^.

Cd is a guest element found in all forms of Zn ores due to geochemical similarities. Hence, during the smelting of Zn, significant amounts of Cd are discharged into the environment. In the manufacturing of Zn, Cd-rich dust is discharged into the environment, has a short retention time, and is deposited locally^39,40^. Although the Lohagara sub-district in Narail, Bangladesh, is a rural area, Cd concentrations there were comparable to those at urban sites. Cd is one of the most mobile heavy metals in the environment, but its bioavailability is complex^41^. However, the Cd pollution in the rural area also raised questions about the presence of high levels of Cd in the soil and vegetation of Bangladesh naturally or by weathering continental slopes like As. Furthermore, several studies indicated that some of the main sources of Cd exposure in Bangladesh are tobacco smoking, food including cereals, vegetables and mainly accumulation by rice.

Diet is most likely the main source of Cd exposure in Bangladesh for those who live in rural environments with essentially no high industrial emissions^12,42–44^. Rice can bioaccumulate Cd, resulting in an elevated concentration, in locations with both geogenic and anthropogenic elevated Cd concentrations in soil and groundwater. Potential causes include the production of rice with a higher affinity for Cd retention, the use of excessive nitrogen fertilisers, pollution from irrigation and air deposition, more acidic soils, etc^41^.

Vehicular emissions, battery recycling, industrial discharges, construction, and paints are the primary contributors to Pb contamination. Although leaded gasoline has been phased out, older vehicles and two-stroke engines can still release lead into the environment. Informal and unregulated recycling of lead–acid batteries is prevalent in many urban areas, especially in Dhaka. This process releases significant amounts of lead into the air and soil. In this study, Pb pollution was found to be significantly higher in the urban industrial area because the area is characterised by a lot of toxic chemical disposal from textile factories, domestic waste, vehicles, electronic belongings maintenance shops, and manufacturers of artificial cosmetics. Since 2000, the Bangladesh Government has decided to provide unleaded gasoline, and the concentration of Pb has decreased to some extent in the environment.

Furthermore, the main source of Pb in road dust is the use of substandard enamel and distemper paints in developing nations’ cities like Dhaka. Vibrant glazes on ceramic kitchenware often cause heavy metals pollution like Pb or Cd^28^. In this study, Pb pollution was found to be significantly higher in Zinzira Palace Rd, Boro Katara Lane, and near the Ambagicha High School of the South Keraniganj commercial and industrial area than in the other areas of Dhaka city. Near the Ambagicha High School site, the metallic concentration was the highest. This mainly arose from the garbage dumping sites. Zinzira is a highly gathered industrial area characterised by a lot of toxic chemical disposal from textile factories, domestic waste, vehicles, electronic belongings maintenance shops, and artificial cosmetics manufacturing. The findings were similar to the other industrial area in Tongi, Bangladesh^45^. In Dhaka, Bangladesh, air Pb concentrations were noted to be extremely high between 1997 and 2000^46^. In the year 2000, the Bangladesh Government decided to provide unleaded gasoline, and the concentration of Pb decreased to some extent in the environment.

Nowadays, many uncontrolled factors are contributing significantly to the case of Pb emission to the environment. The most frequent source of Pb exposure in urban areas is transportation^47^. The use of power-based auto rickshaws has increased during the past ten years, both in the urban and rural areas of Bangladesh. The haphazard recycling of used Pb–acid batteries across the country is a serious issue of metal pollution in the environment^31^. Because open-pit battery recycling is so common in the city, a lot of Pb is released into the road dust, which accumulates in the roadside soil. Many industries, including ceramic factories, battery production and processing factories, plastic manufacturing factories, chemical and pharmaceutical factories, cement production and processing factories, fertiliser factories, metal workshops, and car repair and welding workshops, are reliable sources of Pb in Bangladesh’s major cities, particularly in the industrial area. Vibrant glazes on ceramic kitchenware often include heavy metals like Pb or Cd^48^.

In the rural area, Cd concentrations were comparable to those at urban sites. Cd pollution in agricultural soils and its subsequent transmission to crops is a significant environmental and public health problem of great worldwide concern, particularly in densely populated developing nations such as Bangladesh^49^. Several studies indicate that one of the main sources of Cd exposure in Bangladesh is tobacco smoking, food including cereals, vegetables, and seafood. Diet is most likely the main source of Cd exposure in Bangladesh for those who live in rural environments with essentially no high industrial emissions^12,42–44^. In addition, the ongoing mega projects for constructing bridges, railways, and infrastructures prove that the source of the high levels of PM2.5 and PM10 was the mobility of the contaminated road dust from urban to rural areas. Overall, Cd and Pb pollution was also found to be higher in the other research related to Bangladesh, like other polluted cities in the world^6,11,35,50^.

Cr and Ni are released from the anthropogenic activity processes, and the main sources are intensive fertilisation, fossil fuels or coal-burning emissions, leather factories and tanneries, cement factories, vehicle emissions, manufacturing and construction activities, and activities of mining and smelting^51^. The high concentration of metals in the roadside dust and soil can pose a high health risk to children and adults. Urban inhabitants and people walking on the roadways are negatively impacted by heavy metals’ major contribution to urban diffuse pollution in developing countries like Bangladesh^52^. They may accumulate in the body’s fatty tissues and have an impact on the neurological system^53^.

## Conclusion

We found that Cd and Pb were the main pollutants for both roadside soil and roadside perennial trees. Cd pollution was also dominant in rural areas. The analysis of all heavy metal indices, such as the geo-accumulation index, contamination factor, and ecological risk assessment, indicated a higher concentration of Cd. According to the effect of single metal toxicity, Cd was the main source of ecological risk, and all the sampling locations had extremely high ecological risk (Er > 160); more than 80% of the ecological risk resulted in Cd pollution. In this study, we found that *Ficus benghalensis* and *Polyalthia longifolia* were good indicators for Pb accumulation. The PCA indicated that Cd, Cr, Ni, and Pb mostly derived from anthropogenic sources. The multiple novel approaches and overall findings of this study can inform better indicators of pollution assessment in Bangladesh and contribute to taking the necessary remedial measures to mitigate pollution.

### Methods

#### Study areas

**Fig. 9:**
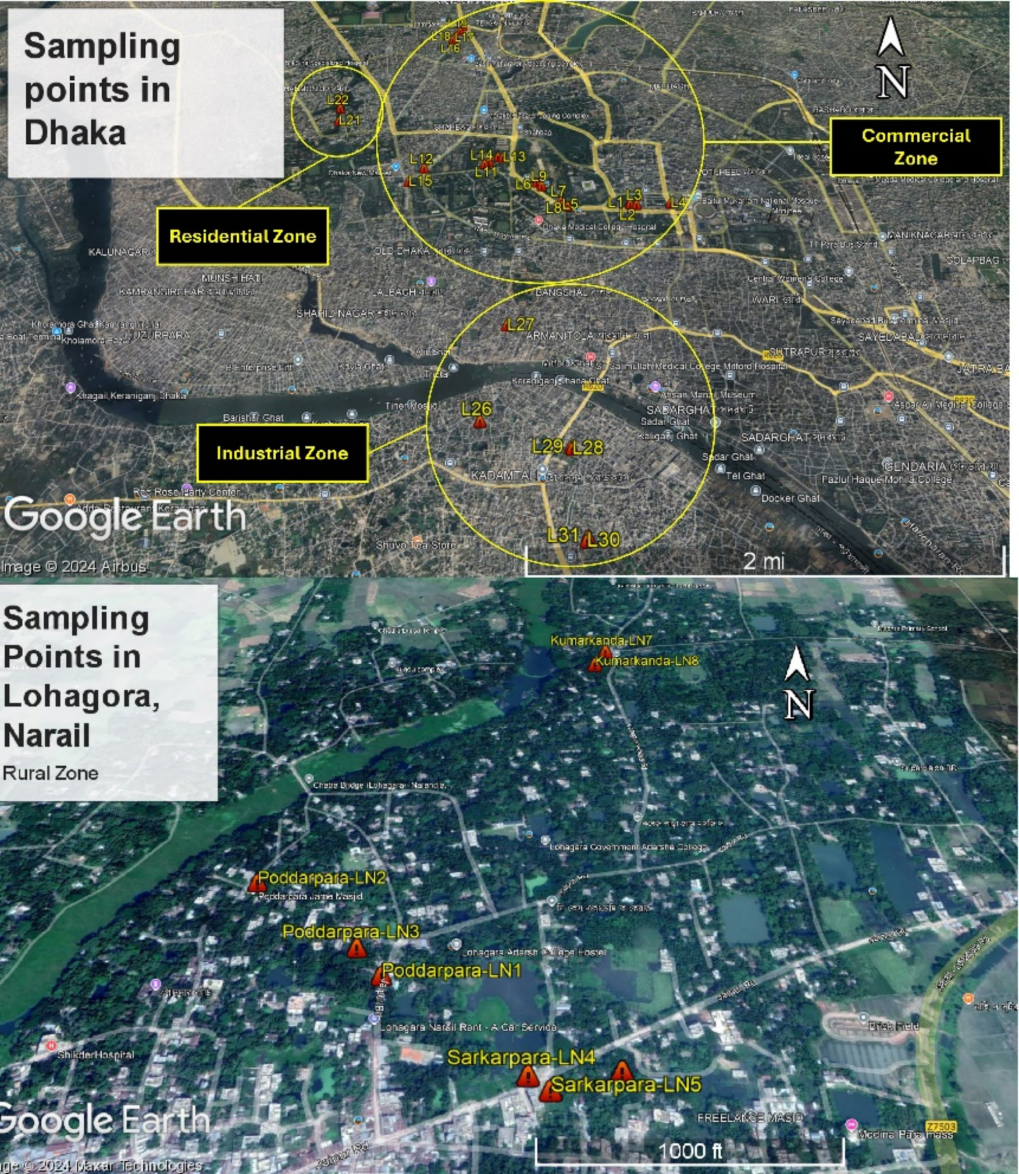
Map of the studied region and the sampling areas in Dhaka Metropolitan area, Banglades and rural area in Lohagara sub-district, Narail, Bangladesh (created using Google Earth Pro and ArcGIS 10.8).

Roadside topsoil and tree leaf samples were collected from 39 sites of the Dhaka South City Corporation and 8 sites of the Lohagara sub-district, Narail, Bangladesh. Dhaka is the capital of Bangladesh, one of the most densely populated regions of the world. The city is located in the central part of Bangladesh (23°42′N and 90°22′E) and serves as the political, economic, and cultural hub of the country. Lohagara Upazila (Narail district) is located between 23°05’ and 23°19’ north latitudes and between 89°29’ and 89°46’ east longitudes of Bangladesh. There were four kinds of sampling locations: commercial areas (the University of Dhaka and adjacent locations), residential areas (Dhanmondi area), industrial areas (South Keraniganj), and rural areas (Lohagara sub-district, Narail, Bangladesh; Fig. 9; Table S6). We collected our samples from January to February when the season was dry, and the air remained much more polluted in Bangladesh. Dhaka undergoes four distinct seasons: a dry winter (December–February), a hot pre-monsoon summer (March–May), a wet monsoon (June–September), and a post-monsoon autumn (October–November). Summer temperatures reach 38–41℃, but January averages range from 16℃ to 20 °C. The city’s land utilisation comprised residential (44.35%), commercial (4.29%), and industrial zones (2.01%), alongside substantial green spaces and transit infrastructure. Extended preceding dry-weather intervals during winter may exacerbate heavy metal contamination and ecological hazards^13^.

## Sample collection

Three species (*Ficus benghalensis* L. 1753, *Swietenia macrophylla* K. 1886, and *Polyalthia longifolia* Sonn. 1864) were selected for this study and the formal identification of the plant material was made by Uddin and Hassan. These species are accessible for study and representative of both urban and rural roadside vegetation because they are frequently found along roadsides in Bangladesh and other South Asian countries^54–56^. The *F. benghalensis* is one of the most dominant and abundant species in Asia and its leaves is rich in flavonoids, phenols, terpenoids which is used as Ayurvedic remedies^54^. *Swietenia macrophylla* is the most important tree in the aspect of economy in many neotropical countries. This species is also used as medicine because of its antimicrobial, antioxidants effects^55^. *Polyalthia longifolia* is native medicinal plants in the tropical and subtropical regions. Therapeutic applications are used for anticancer, antimicrobial and hypotensive acitivities^56^. These trees are prevalent in public areas and offer shade and shelter, so understanding their interaction with pollutants is important for urban planning and.

public health. The age of individuals was about 5 to 6 years, the state of the trees was healthy, and the presence of pests and symptoms of disease were not found by visual analysis. We randomly chose three individuals. In the case of each species from the external and internal parts of trees 7–8 leaves were collected at a 1.1.5 m height. At each sampling area, five soil samples were collected from a depth of 0–20 cm with a small hand spade. After collection, leaves and soil samples were dried at room temperature until the laboratory process was completed.

### Laboratory analysis of samples

The leaf samples were cleaned with tap water because using distilled water may have resulted in significant changes in the elemental concentrations of leaves due to osmotic effects^9,37^. Leaf samples were dried for 24 h at 60℃ (WTE Binder ED 53 drying oven) and then homogenised using a homogeniser (Retsch Knife Mill GRINDOMIX GM 200). Before pre-treatment, the homogenised leaf samples were kept in plastic tubes. Soil samples were dried at 105℃ in the drying machine. Following drying, with a plastic tweezer, small brick fragments, plant roots, and other leftovers were removed. After that, a 2 mm plastic sieve was used to filter the samples. Before pre-treatment, the soil samples were homogenised with an agate mortar and pestle and kept in plastic tubes (Fig. S1). For elemental analysis, 0.1 g of leaf tissue and 0.1 g of soil were digested using a heating burner and 10 ml of 65% (m/m) HNO_3_ and 0.2 ml of 30% (m/m) H_2_O_2_. The elemental concentrations of Cd, Ni, Pb, and Cr were analysed using inductively coupled plasma optical emission spectrometry (ICP-OES 5110 Agilent Technologies). For the analysis, a six-point calibration procedure was used based on a multi-element calibration solution (Merck ICP multi-element standard solution IV; Fig. S2). Quality control was carried out with CRM reference materials SQC001-30G and 1547. Recoveries were within ± 10% of the certified values. Soil (SQC001-30G) and peach leaves (1547) CRM were used, and the recoveries were within ± 10% of the certified values. Table S7).

### Pollution indices

Pollution indices are useful tools for a comprehensive evaluation of soil contamination. They can be of great importance in the assessment of environmental quality and the prediction of health risks. The geo-accumulation index (I_geo_) CF, pollution load index, potential ecological risk factor, the risk index, and bioaccumulation factor of leaves were calculated (Table S8)^55–59^. The equations of the indices are shown in Table S8. In this study, the United States of America’s Environmental Protection Agency Ecological Soil Screening Levels (US EPA Eco-SSLs) of topsoil values were considered as background values^29,30^. Eco-SSLs refer to concentrations of pollutants in soil that are designed to mitigate risks to ecological receptors. The distribution of Eco-SSLs by the US EPA refers to a range of inorganic and organic pollutants commonly encountered in soil within Superfund sites. Eco-SSLs are employed for the purpose of assessing ecological hazards at sites that have been contaminated, as well as for the establishment of remediation objectives^41–43^.

### Statistical analysis

SPSS Statistics 25 and PAST 4.03 statistical packages were used during the statistical analyses. The normal distribution was tested with a Shapiro–Wilk test. The homogeneity of variances was tested using Levene’s test. PCA was used to study the interaction between tree species and urbanisation on the elemental concentration of topsoil and leaves. The elemental concentration of samples and the studied areas were compared with a two-way ANOVA where one factor was the studied areas and the other factor was tree species. As a post hoc test, the LSD Multiple Comparison test was used to explore the significant differences. In a study area map using ArcGIS 10.8, the inverse distance weighting (IDW) interpolation method was employed to generate a continuous surface representation of the pollution levels across the study area. IDW is an interpolation method used in geographic information system (GIS) software like ArcGIS to estimate values at unsampled locations based on the values available at sampled locations. This approach has been widely used in similar environmental studies where spatial variability is high, and localized sources influence concentration patterns. IDW effectively captures the spatial structure of heavy metal distribution in areas with localized contamination, making it an appropriate choice for our dataset.

## Electronic supplementary material

Below is the link to the electronic supplementary material.

Supplementary Material 1

## Data Availability

Data are available from the corresponding author on reasonable request.

## References

[1] Rahman, M. S., Kumar, S., Nasiruddin, M. & Narottam, S. Deciphering the origin of Cu, Pb and Zn contamination in school dust and soil of Dhaka, a megacity in Bangladesh. *Environ. Sci. Pollut. Res.* **28**, 40808–40823. 10.1007/s11356-021-13565-7 (2021). 10.1007/s11356-021-13565-7 33772469

[2] Ahmed, F. et al. Spatial distribution and source identification of heavy metal pollution in roadside surface soil: a study of Dhaka Aricha highway, Bangladesh. *Ecol. Process.* **5**, 2. 10.1186/s13717-016-0045-5 (2016).

[3] Kormoker, T. et al. Road dust–driven elemental distribution in megacity Dhaka, Bangladesh: environmental, ecological, and human health risks assessment. *Environmental Science and Pollution Re*search 29, 22350–22371 (2022). 10.1007/s11356-021-17369-7 10.1007/s11356-021-17369-7 34782979

[4] Rahman, M. S. et al. Assessing risk to human health for heavy metal contamination through street dust in the Sou, theast Asian megacity: Dhaka, Bangladesh. *Sci. Total Environ.* **660**, 1610–1622. 10.1016/j.scitotenv.2018.12.425 (2019). 30743952 10.1016/j.scitotenv.2018.12.425

[5] Guan, Z. H., Li, X. G. & Wang, L. Heavy metal enrichment in roadside soils in the eastern Tibetan Plateau. *Environmental Science and Pollution Re*search 25, 7625–7637 (2018). 10.1007/s11356-017-1094-8 10.1007/s11356-017-1094-8 29285695

[6] Sultan, M. B., Choudhury, T. R., Alam, M. N., Doza, M. B. & Rahman, M. M. Soil, dust, and leaf-based novel multi-sample approach for urban heavy metal contamination appraisals in a megacity, Dhaka, Bangladesh. *Environ. Adv.* **7**, 100154. 10.1016/j.envadv.2021.100154 (2022).

[7] Kolakkandi, V. et al. Spatially resolved distribution, sources and health risks of heavy metals in size-fractionated road dust from sites across megacity Kolkata, India. *Science of Total Environment* 705, 135805 (2019). https://doi.org/10. 1016/j. scito tenv. 2019. 135805. 10.1016/j.scitotenv.2019.135805 31972942

[8] Canli, M. & Atli, G. The relationships between heavy metal (Cd, Cr, Cu, Fe, Pb, Zn) levels and the size of six mediterranean fish species. *Environ. Pollut.* **121**, 129–136. 10.1016/S0269-7491(02)00194-X (2003). 12475070 10.1016/s0269-7491(02)00194-x

[9] Grzebisz, W., Ciesla, L., Komisarek, J. & Potarzycki, J. Geochemical assessment of heavy metals pollution of urban soils. *Pol. J. Environ. Stud.* **11**, 493–499 (2000).

[10] Roy, S., Gupta, S. K., Prakash, J., Habib, G. & Kumar, P. A global perspective of the current state of heavy metal contamination in road dust. *Environ. Sci. Pollut. Res. Int.* **29**, 33230–33251. 10.1007/s11356-022-18583-7 (2022). 35022986 10.1007/s11356-022-18583-7

[11] Zakir, H. M., Sultana, N. & Akter, M. Heavy metal contamination in roadside soils and grasses: a case study from Dhaka City, Bangladesh. *J. Chem. Biol. Phys. Sci.* **4**, 1661–1673 (2014).

[12] Islam, M. M., Karim, M. R., Zheng, X. & Li, X. Heavy metal and metalloid pollution of soil, water and foods in Bangladesh: A critical review. *Int. J. Environ. Res. Public Health*. **15**, 2825. 10.3390/ijerph15122825 (2018). 30544988 10.3390/ijerph15122825 PMC6313774

[13] Kabir, M. H., Wang, Q., Rashid, M. H., Wang, W. & Isobe, Y. Assessment of bioaccessibility and health risks of toxic metals in roadside dust of Dhaka City, Bangladesh. *Atmosphere* **13**, 488. 10.3390/atmos13030488 (2022).

[14] Ahmad, J. U. & Goni, M. A. Heavy metal contamination in water, soil, and vegetables of the industrial areas in Dhaka, Bangladesh. *Environ. Monit. Assess.* **166**, 347–357. 10.1007/s10661-009-1006-6 (2010). 19521788 10.1007/s10661-009-1006-6

[15] Mottalib, M. A., Somoal, S. H., Aftab, M., Shaikh, A. & Islam, M. S. Heavy metal concentrations in contaminated soil and vegetables of tannery area in Dhaka, Bangladesh. *Int. J. Curr. Res.* **8**, 30369–30373 (2016).

[16] Shaheen, N. et al. Presence of heavy metals in fruits and vegetables: health risk implications in Bangladesh. *Chemosphere* **152**, 431–438. 10.1016/j.chemosphere.2016.02.060 (2016). 27003365 10.1016/j.chemosphere.2016.02.060

[17] Ahmed, M. K. et al. Human health risks from heavy metals in fish of Buriganga river, Bangladesh. *Springerplus* **5**, 1697. 10.1186/s40064-016-3357-0 (2016). 27757369 10.1186/s40064-016-3357-0 PMC5047865

[18] Islam, F. et al. Heavy metals in water, sediment and some fishes of Karnofuly river, Bangladesh. *Int. J. Environ. Res.* **4**, 321–332 (2013).

[19] Simon, E., Molnár, V. É., Tóthmérész, B. & Szabó, S. Ecological assessment of particulate material (PM5 and PM10) in urban habitats. *Atmosphere* **11**, 559. 10.3390/atmos11060559 (2020).

[20] Molnár, V. É., Simon, E., Ninsawat, S., Tóthmérész, B. & Szabó, S. Pollution assessment based on element concentration of tree leaves and topsoil in Ayutthaya Province, Thailand. *Int. J. Environ. Res. Public Health*. **17**, 5165. 10.3390/ijerph17145165 (2020). 32708947 10.3390/ijerph17145165 PMC7400151

[21] Shahrukh, S. et al. Air pollution tolerance, anticipated performance, and metal accumulation indices of four evergreen tree species in Dhaka, Bangladesh. *Curr. Plant. Biology*. **35–36**10.1016/j.cpb.2023.100296 (2023).

[22] El-Amier, Y. A. & Alghanem, S. M. Tree leaves as bioindicator of heavy metal pollution from soil and ambient air in urban environmental. *Plant. Archives*. **18**, 2559–2566 (2018).

[23] da Ferreira, C., Braga, R. L. & do Nascimento, D. G. Biochar improves growth and physiology of *Swietenia macrophylla* King in contaminated soil by copper. *Sci. Rep.* **14**, 22546. 10.1038/s41598-024-74356-x (2024). 39343801 10.1038/s41598-024-74356-x PMC11439936

[24] Putri, F. A., Tohir, D., Batubara, I. & Asoka, S. F. Active compounds in broadleaf Mahogany (Swietenia macrophylla) seeds as antiaging agent based on molecular Docking study. *Al-Kimia* **11**, 68–81 (2023).

[25] Kabir, M. H. et al. Determination of heavy metal contamination and pollution indices of roadside dust in Dhaka City. *Bangladesh Processes*. **9**, 1732. 10.3390/pr9101732 (2021).

[26] Rahman, S. M. et al. Elemental analysis in surface soil and dust of roadside academic institutions in Dhaka City, Bangladesh, and their impact on human health. *Environ. Chem. Ecotoxicol.* **3**, 197–208. 10.1016/j.enceco.2021.06.001 (2021).

[27] WHO-Europe Health-risk of heavy metals from long range transboundary air pollution. Copenhagen, Denmark: Regional Office for Europe & Joint WHO/Convention Task Force on the Health Aspects of Air Pollution. (2007). Retrieved from https://apps.who.int/iris/handle/10665/107872

[28] Nawaz, R. et al. Air pollution tolerance index and heavy metals accumulation of tree species for sustainable environmental management in megacity of Lahore. *Air* **1**, 55–68. 10.3390/air1010004 (2022).

[29] US EPA (United States Environmental Protection Agency). *Risk Assessment Guidance for Superfund. Volume I. Human Health Evaluation Manual* (Part E, 2002). Supplemental Guidance for Dermal Risk Assessment).

[30] US EPA (United States Environmental Protection Agency). Human Health Evaluation Manual, Supplemental Guidance: Standard Default Exposure Factors. Washington, DC, USA. (1991).

[31] Nargis, A. et al. Source identification, contamination status and health risk assessment of heavy metals from road dusts in Dhaka, Bangladesh. *J. Environ. Sci. (China)*. **121**, 159–174. 10.1016/j.jes.2021.09.011 (2022). 35654507 10.1016/j.jes.2021.09.011

[32] Perumal, K. A. Heavy metal pollutants and their Spatial distribution in surface sediments from Thondi Coast, Palk Bay, South India. *Environ. Sci. Europe*. **33**10.1186/s12302-021-00501-2 (2021).

[33] Mudgal, V., Madaan, N., Mudgal, A., Singh, R. B. & Mishra, S. Effect of toxic metals on human health. *Open. Nutraceuticals J.* **3**, 94–99 (2010).

[34] Peng, L. et al. W.Comprehensive Urumqi screening for potentially toxic metals in soil-dust-plant total environment and evaluation of children’s (0–6 years) risk-based blood lead levels prediction. *Chemosphere* **258**, 127342. 10.1016/j.chemosphere.2020.127342 (2020). 32947679 10.1016/j.chemosphere.2020.127342

[35] Kabir, M. H. et al. A comprehensive assessment of heavy metal contamination in road dusts along a hectic National highway of Bangladesh: Spatial distribution, sources of contamination, ecological and human health risks. *Toxin Reviews*. **41**, 860–879. 10.1080/15569543.2021.1952436 (2022).

[36] Xia, X., Chen, X., Liu, R. & Liu, H. Heavy metals in urban soils with various types of land use in Beijing, China. *J. Hazard. Mater.* **186**, 2043–2050. 10.1016/j.jhazmat.2010.12.104 (2011). 21242029 10.1016/j.jhazmat.2010.12.104

[37] Manta, D. S., Angelone, M., Bellanca, A., Neri, R. & Sprovieri, M. Heavy metals in urban soils: a case study from the City of Palermo (Sicily), Italy. *Sci. Total Environ.* **300**, 229–243. 10.1016/s0048-9697(02)00273-5 (2002). 12685485 10.1016/s0048-9697(02)00273-5

[38] Men, C. et al. Pollution characteristics, risk assessment, and source apportionment of heavy metals in road dust in Beijing, China. *Sci. Total Environ.* **612**, 138–147 (2018). 28850834 10.1016/j.scitotenv.2017.08.123

[39] Bi, Z., Qiao, S., Zhou, J., Tang, X. & Cheng, Y. Inhibition and recovery of anammox biomass subjected to short-term exposure of cd, Ag, hg and Pb. *Chem. Eng. J.* **244**, 89–96. 10.1016/j.cej.2014.01.062 (2014).

[40] Zheng, J. et al. Heavy metals in food, house dust, and water from an e-waste recycling area in South China and the potential risk to human health. *Ecotoxicol. Environ. Saf.* **96**, 205–212. 10.1016/j.ecoenv.2013.06.017 (2013). 23849468 10.1016/j.ecoenv.2013.06.017

[41] Kubier, A., Wilkin, R. T. & Pichler, T. Cadmium in soils and groundwater: A review. *Appl. Geochem.* **108**, 104388. 10.1016/j.apgeochem.2019.104388 (2019). 10.1016/j.apgeochem.2019.104388 PMC7147761 32280158

[42] Moynihan, M. et al. Dietary predictors of urinary cadmium among pregnant women and children. *Sci. Total Environ.* **575**, 1255–1262. 10.1016/j.scitotenv.2016.09.204 (2017). 27707662 10.1016/j.scitotenv.2016.09.204 PMC5433527

[43] Kippler, M. et al. Environmental exposure to arsenic and cadmium during pregnancyand fetal size: a longitudinal study in rural Bangladesh. *Reprod. Toxicol.* **34**, 504–511 (2012). https://www.sciencedirect.com/science/article/pii/S0890623812002845 22985739 10.1016/j.reprotox.2012.08.002

[44] Hossain, S., Latifa, G. A., Al Nayeem, A. & Prianqa & Review of cadmium pollution in Bangladesh. *J. Water Health*. **9**, 190913. 10.5696/2156-9614-9.23.190913 (2019). 10.5696/2156-9614-9.23.190913 PMC6711336 31497376

[45] Mitra, A. K. et al. Lead poisoning: an alarming public health problem in Bangladesh. *Int. J. Environ. Res. Public Health*. **6**, 84–95. 10.3390/ijerph6010084 (2009). 19440271 10.3390/ijerph6010084 PMC2672336

[46] Biswas, T., Garnett, S. P., Pervin, S. & Rawal, L. B. The prevalence of underweight, overweight and obesity in Bangladeshi adults: data from a National survey. *PLoS One*. **16**, e0177395. 10.1371/journal.pone.0177395 (2017). 10.1371/journal.pone.0177395 PMC5433717 28510585

[47] Delibašić, Š. Đ.-K. Health risk assessment of heavy metal contamination in street dust of federation of Bosnia and Herzegovina. *Hum. Ecol. Risk Assess.* **27**, 1296–1308 (2020).

[48] Aderemi, T. A., Adenuga, A. A. & Oyekunle, J. A. O. High level leaching of heavy metals from colorful ceramic foodwares: a potential risk to humans. *Environ. Sci. Pollut. Res.* **24**, 17116–17126. 10.1007/s11356-017-9385-7 (2017). 10.1007/s11356-017-9385-7 28585010

[49] Al Mamun, A., Sarker, P., Rahaman, M. S., Kabir, M. M. & Maruo, M. Evaluation of contamination and accumulation of heavy metals in the Dhaleswari river sediments. *Bangladesh Int. J. Environ.* **10**, 1–19 (2021).

[50] Ahmed, F. & Ishiga, H. Trace metal concentrations in street dusts of Dhaka City, Bangladesh. *Atmos. Environ.* **40**, 3835–3844. 10.1016/j.atmosenv.2006.03.004 (2006).

[51] Lwin, C. S., Seo, B. H., Kim, H. U., Owens, G. & Kim, K. R. Application of soil amendments to contaminated soils for heavy metal immobilization and improved soil quality—a critical review. *Soil. Sci. Plant. Nutr.* **64**, 156–167. 10.1080/00380768.2018.1440938 (2018).

[52] Pal, S. K., Wallis, S. G. & Arthur, S. An assessment of heavy metals pollution potential of road sediment derived from a suburban road network under different weather conditions. *Environ. Eng. Manag. J.* **17**, 1955–1966 (2018).

[53] Hassanien, M. A. & Shahawy, A. M. E. Environmental heavy metals and mental disorders of children in developing countries. In: Simeonov, L., Kochubovski, M., Simeonova, B. (eds) Environmental heavy metal pollution and effects on child mental development. nato science for peace and security series c: environmental security. Dordrecht, Netharlands: Springer, Dordrecht, (2011). 10.1007/978-94-007-0253-0_1 (2010).

[54] Murugesu, S., Selamat, J. & Perumal, V. Phytochemistry, Pharmacological properties, and recent applications of *Ficus benghalensis* and *Ficus religiosa*. *Plants* **10**, 2749. 10.3390/plants10122749 (2021). 34961220 10.3390/plants10122749 PMC8707271

[55] Moghadamtousi, S. Z., Goh, B. H., Chan, C. K., Shabab, T. & Kadir, H. A. Biological activities and phytochemicals of *Swietenia macrophylla* King. *Molecules* **18**, 10465–10483. 10.3390/molecules180910465 (2013). 23999722 10.3390/molecules180910465 PMC6270564

[56] Katkar, K. V., Suthar, A. C. & Chauhan, V. S. The chemistry, pharmacologic, and therapeutic applications of polyalthia longifolia. *Pharmacogn. Rev.* **4**, 62–68. 10.4103/0973-7847.65329 (2010). 22228943 10.4103/0973-7847.65329 PMC3249904

[57] Muller, G. Index of geoaccumulation in sediments of the rhine river. *J. Geol.* **2**, 108–118 (1979).

[58] Mavakala, B. M. et al. Evaluation of heavy metal content and potential ecological risks in soil samples from wild solid waste dumpsites in developing country under tropical conditions. *Environ. Challenges*. **7**, 100461. 10.1016/j.envc.2022.100461 (2022).

[59] Ololade, I. A. An assessment of heavy-metal contamination in soils within auto-mechanic workshops using enrichment and contamination factors with geoaccumulation indexes. *J. Environ. Prot.* **5**, 49118. 10.4236/jep.2014.511098 (2014).

