# Supplementary material for: Assessment of anthropogenic activities impact based on metals in soil and tree leaves along roadside in Bangladesh
Source: Sci Rep. 2025 Feb 26;15:6960. doi: 10.1038/s41598-025-91683-9 (PMC11865477; doi:10.1038/s41598-025-91683-9)
Supplement: Supplementary file 1 — Supplementary Material 1 [file 41598_2025_91683_MOESM1_ESM.docx]

**Supplementary Materials**


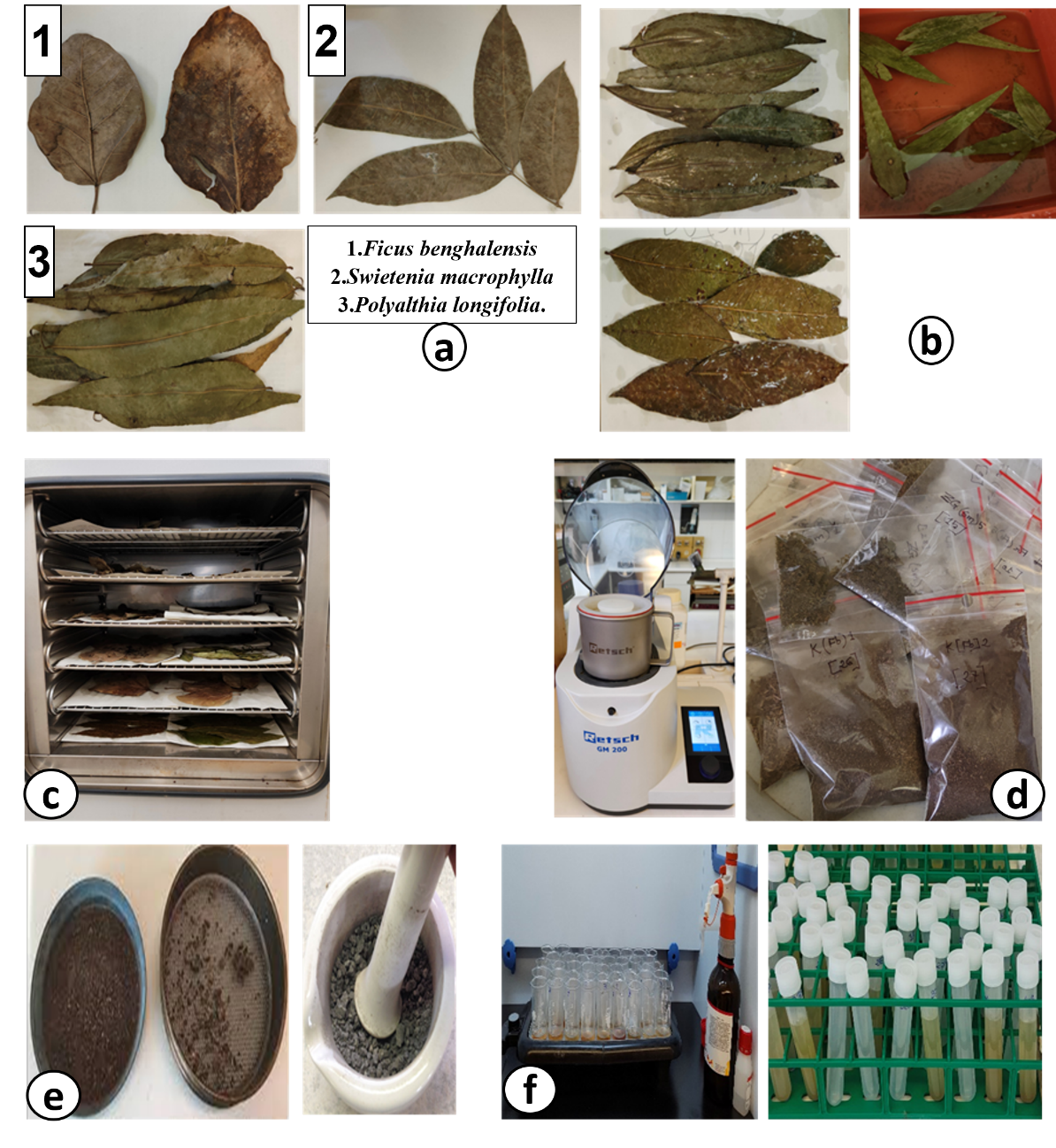


**Figure S1.** Preparation of leaf and soil samples for digestion. (a) Dry leaf samples of F. benghalensis, S. macrophylla, and P. longifolia. (b) washing the leaf samples with tap water. (c) Drying of the leaf samples in the oven. (d) and (e) Homogenization of the leaf samples. (f) Digestion of the soil and leaf samples with Nitric acid and Hydrogen peroxide.
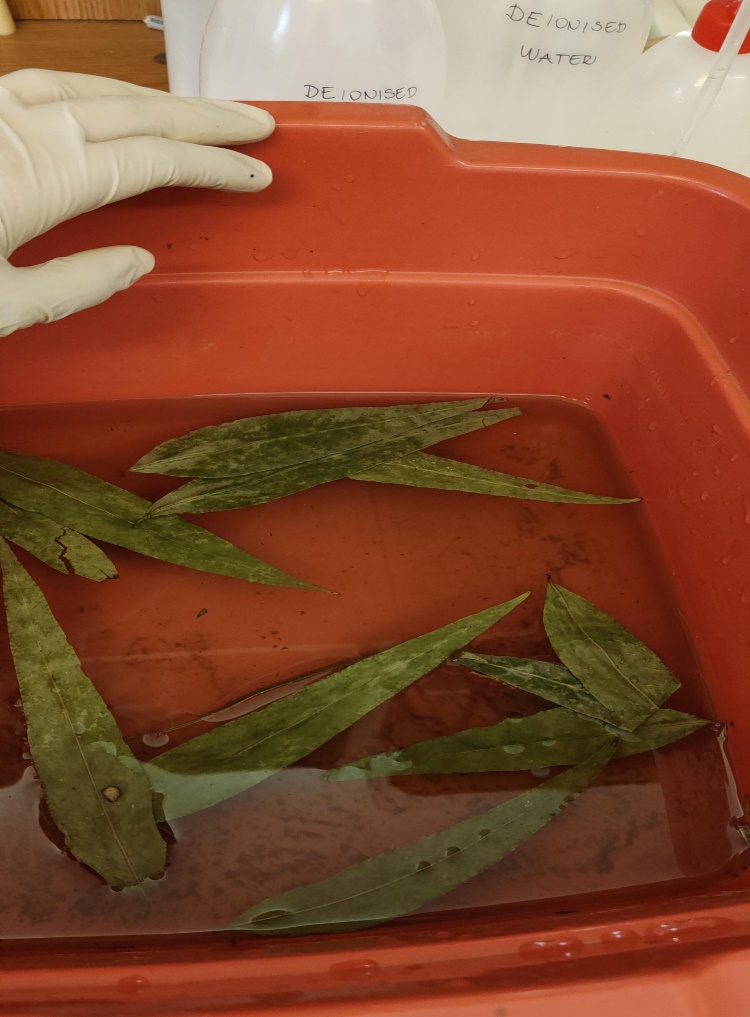


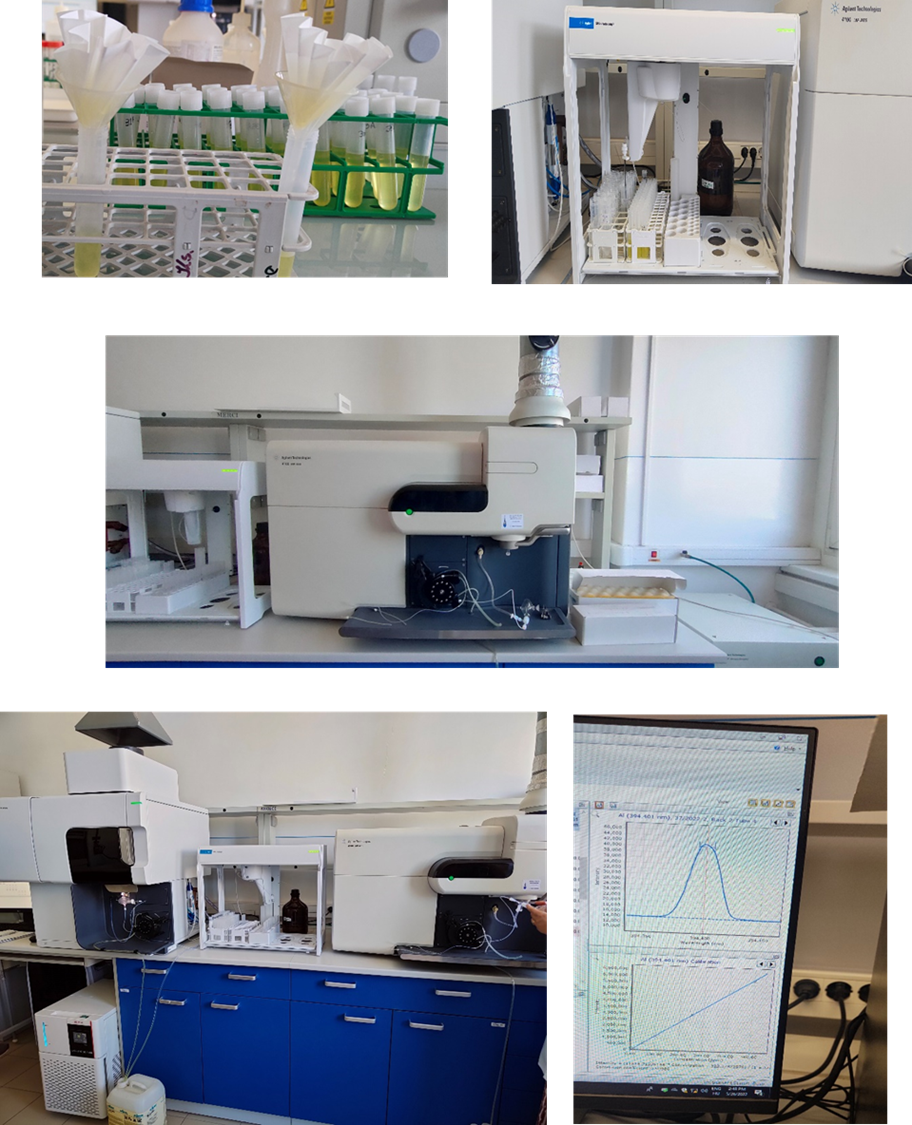


**Figure S2**.Elemental concentration analysis by ICP-OES5110 Agilent Technologies.

**Table S1.** Results of the one-way ANOVA for different heavymetal concentrations in roadside soil in different areas.

| Metals | F | *p* |
| --- | --- | --- |
| Cd | 0.35 | 0.789 |
| Cr | 1.33 | 0.282 |
| Ni | 1.69 | 0.187 |
| Pb | 4.95 | 0.006^*^ |

**Table S2.** Results of the two-way ANOVA for metal concentrations in studied tree leaves in different areas.

| Metals | Source | Type III Sum of Squares | df | Mean Square | F | *p* |
| --- | --- | --- | --- | --- | --- | --- |
| Cd | Intercept | 2.761 | 1 | 2.761 | 118.116 | <0.001 |
|  | area | 0.131 | 3 | 0.044 | 1.872 | 0.156 |
|  | species | 0.000 | 2 | 0.000 | 0.009 | 0.991 |
|  | area * species | 0.052 | 4 | 0.013 | 0.559 | 0.694 |
| Cr | Intercept | 61.505 | 1 | 61.505 | 90.544 | <0.001 |
|  | area | 2.751 | 3 | 0.857 | 1.261 | 0.306 |
|  | species | 0.219 | 2 | 0,11 | 0.161 | 0.852 |
|  | area * species | 2.47 | 4 | 0.617 | 0.909 | 0.472 |
| Ni | Intercept | 202.559 | 1 | 202.559 | 33.529 | <0.001 |
|  | area | 24.376 | 3 | 8.125 | 1.345 | 0.279 |
|  | species | 5.720 | 2 | 2.860 | 0.473 | 0.628 |
|  | area * species | 25.070 | 4 | 6.268 | 1.073 | 0.405 |
| Pb | Intercept | 1635.548 | 1 | 1635.548 | 55.436 | <0.001 |
|  | area | 139.900 | 3 | 46.633 | 1.581 | 0.215 |
|  | species | 64.792 | 2 | 32.396 | 1.098 | 0.347 |
|  | area * species | 31.823 | 4 | 7.965 | 0.270 | 0.895 |


**Table S3.** Value of Igeo for metals in studies sites (mean ± SD).

| Sites | Cd | Cr | Ni | Pb |
| --- | --- | --- | --- | --- |
| commercial | 2.07 ± 0.09 | 0.85 ± 0.50 | 0.62 ± 0.47 | 0.81 ± 0.44 |
| residential | 2.08 ± 0.07 | 0.91 ± 0.33 | 0.80 ± 0.50 | 0.79 ± 0.37 |
| industrial | 2.15 ± 0.40 | 0.78 ± 0.18 | 0.62 ± 0.41 | 1.36 ± 0.17 |
| rural | 2.03 ± 0.05 | 0.62 ± 0.24 | 0.78 ± 0.28 | 0.22 ± 0.25 |

**Table S4.** Value of contamination factor for metals in study sites based on leaves (mean ± SD).

| Sites | Species | Cd | Cr | Ni | Pb |
| --- | --- | --- | --- | --- | --- |
| commercial | *F. benghalensis* | 21 ± 5 | 1 ± 1 | 2 ± 2 | 6 ± 2 |
|  | *P. longifolia* | 17 ± 11 | 1 ±1 | 1 ± 1 | 5 ± 4 |
|  | *S. macrophylla* | 19 ± 6 | 2 ± 1 | 2 ± 2 | 4 ± 2 |
| residential | *P. longifolia* | 15 ± 13 | 1 ± 1 | 1 ± 1 | 2 ±3 |
|  | S. macrophylla | 15 ± 1 | 1 ± 1 | 1 ± 1 | 2 ± 1 |
| industrial | *F. benghalensis* | 12 ± 7 | 1 ± 1 | 1 ± 1 | 5 ± 3 |
|  | *S. macrophylla* | 18 ± 4 | 1 ± 1 | 2 ± 3 | 5 ± 1 |
| rural | *F. benghalensis* | 13 ± 4 | 1 ± 1 | 1 ± 1 | 4 ± 4 |
|  | *P. longifolia* | 14 ± 6 | 1 ± 1 | 1 ± 1 | 2 ± 2 |
|  | *S. macrophylla* | 9 ± 3 | 1 ± 1 | 1 ± 1 | 1 ± 1 |

**Table S5.** Value of ERI for metals in study sites (mean ± SD).

| Sites | Cd | Cr | Ni | Pb |
| --- | --- | --- | --- | --- |
| commercial | 189 ± 12 | 6 ± 3 | 15 ± 5 | 12 ± 5 |
| residential | 192 ± 9 | 6 ± 1 | 16 ± 7 | 13 ± 7 |
| industrial | 174 ± 91 | 4 ± 2 | 11 ± 7 | 14 ± 7 |
| rural | 184 ± 7 | 5 ± 1 | 16 ± 3 | 5 ± 1 |


**Table S6**.Sampling Locations for heavy metal concentration analysis of roadside soil and tree leaves in Bangladesh.

| **No.** | **Longitude** | **Latitude** | **Locations** | **Species** | **Area** |
| --- | --- | --- | --- | --- | --- |
| L1 | 90.406507 | 23.727941 | Abdul Gani Rd | *S. macrophylla* | Commercial  (University of Dhaka and adjacent areas  ) |
| L2 | 90.406543 | 23.727827 | Abdul Gani Rd | *S. macrophylla* |  |
| L3 | 90.407128 | 23.727737 | Abdul Gani Rd | *S. macrophylla* |  |
| L4 | 90.410308 | 23.727923 | Bangabandhu Avenue Rd | *S. macrophylla* |  |
| L5 | 90.40016 | 23.727689 | DU swimming pool | *S. macrophylla* |  |
| L6 | 90.397883 | 23.730719 | Secretariat road | *S. macrophylla* |  |
| L7 | 90.400096 | 23.728209 | Doel Chattor | *P. longifolia* |  |
| L8 | 90.400686 | 23.727554 | Curzon Hall Road | *P. longifolia* |  |
| L9 | 90.398336 | 23.730243 | Suhrawardy Udyan Road | *S. macrophylla* |  |
| L10 | 90.394155 | 23.734315 | Madhur Canteen Road | *P. longifolia* |  |
| L11 | 90.386961 | 23.732655 | Social Science Square, DU | *S. macrophylla* |  |
| L12 | 90.394605 | 23.734191 | Aamtola, DU | *S. macrophylla* |  |
| L13 | 90.393267 | 23.73358 | Mukti o Gonotonro Toron | *S. macrophylla* |  |
| L14 | 90.392835 | 23.733277 | Nilkhet Road | *P. longifolia* |  |
| L15 | 90.385586 | 23.730778 | Azimpur Road | *P. longifolia* |  |
| L16 | 90.388757 | 23.754475 | Green Road | *F. benghalensis* |  |
| L17 | 90.388932 | 23.754833 | Green Road | *F. benghalensis* |  |
| L18 | 90.38901 | 23.755038 | Green Road | *F. benghalensis* |  |
| L19 | 90.389544 | 23.756167 | Green Road | *F. benghalensis* |  |
| L20 | 90.389935 | 23.757081 | Near Hossain Tower, Green Road | *F. benghalensis* |  |
| L21 | 90.3776597 | 23.739836 | Dhanmondi Lake Road | *P. longifolia* | Residental |
| L22 | 90.377491 | 23.741828 | Dhanmondi Lake Road | *P. longifolia* |  |
| L23 | 90.377388 | 23.742105 | Dhanmondi Lake Road | *P. longifolia* |  |
| L24 | 90.377265 | 23.743285 | Dhanmondi Lake Road Bridge | *P. longifolia* |  |
| L25 | 90.377155 | 23.743575 | Dhanmondi Lake Road Bridge | *P. longifolia* |  |
| L26 | 90.393 | 23.7054 | Zinzira Palace | *F. benghalensis* | Industrial |
| L27 | 90.395 | 23.714 | Boro Katara Lane | *F. benghalensis* |  |
| L28 | 90.3997 | 23.7032 | Ambagicha High School | *P. longifolia* |  |
| L29 | 90.3996 | 23.7033 | Ambagicha High School | *P. longifolia* |  |
| L30 | 90.4003 | 23.6966 | Chunkutia Girl’s High School | *S. macrophylla* |  |
| L31 | 90.4004 | 23.6969 | Jhaubari Road | *S. macrophylla* |  |
| LN1 | 89.65208 | 23.18986 | Poddar Para | *F. benghalensis* | Rural  (Lohagara sub-district, Narail) |
| LN2 | 89.65043 | 23.1912 | Poddar Para | *F. benghalensis* |  |
| LN3 | 89.65176 | 23.19023 | Poddar Para | *P. longifolia* |  |
| LN4 | 89.65369 | 23.18867 | Sarkar Para | *S. macrophylla* |  |
| LN5 | 89.65391 | 23.18851 | Sarkar Para | *S. macrophylla* |  |
| LN6 | 89.65465 | 23.18872 | Sarkar Para | *S. macrophylla* |  |
| LN7 | 89.65514 | 23.19619 | Kumar Kanda | *P. longifolia* |  |
| LN8 | 89.65493 | 23.19582 | Kumar Kanda | *P. longifolia* |  |

**Table S7.** Results of CRM materials (mg kg-1, mean + SD)

| Elements | Soil | | Leaves | |
| --- | --- | --- | --- | --- |
|  | Reference | Our results | Reference | Our results |
| Cd | 276 ± 7 | 294 ± 5 | 0.026 ± 0.002 | 0.025 ± 0.001 |
| Cr | 277 ± 7 | 286 ± 12 | n.d. | 0.164 ± 0.005 |
| Pb | 228 ± 5 | 242 ± 8 | 0.869 ± 0.018 | 0.980 ± 0.004 |
| Ni | 312 ± 7 | 306 ± 15 | 0.689 ± 0.095 | 0.723 ± 0.002 |

**Table S8.** The used indices

| Name of index | Equation | References |  |
| --- | --- | --- | --- |
| Geoaccumulation Index | 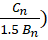 | Muller, 1979 |  |
|  |  |  |  |
| Potential Ecological Risk Factor and the Risk Index | Eri = Tri Cfi | Mavakala et al. 2022 |  |
|  |  |  |  |
|  |  |  |  |
| Contamination Factor | 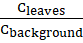 | Ololade, 2014 |  |
|  |  |  |  |
|  |  |  |  |

References

Muller, G. (1979). Index of Geoaccumulation in Sediments of the Rhine River. J Geol 2, 108-118.

Mavakala, B.M., Sivalingam, P., Laffite A:, Mulaji C.K., Giuliani G., Mpiana P.T., Poté J. Evaluation of heavy metal content and potential ecological risks in soil samples from wild solid waste dumpsites in developing country under tropical conditions. Environ Chall 7, 100461. https://doi.org/10.1016/j.envc.2022.100461Ololade, I.A. (2014). An assessment of heavy-metal contamination in soils within auto-mechanic workshops using enrichment and contamination factors with geoaccumulation indexes. J Environ Prot 5, 49118. DOI:10.4236/jep.2014.511098
